# Regional cerebral tau predicts decline in everyday functioning across the Alzheimer’s disease spectrum

**DOI:** 10.1186/s13195-023-01267-w

**Published:** 2023-07-05

**Authors:** Mark A. Dubbelman, Kayden J. Mimmack, Emily H. Sprague, Rebecca E. Amariglio, Patrizia Vannini, Gad A. Marshall, for the Alzheimer’s Disease Neuroimaging Initiative

**Affiliations:** 1 Department of Neurology, Massachusetts General Hospital, Harvard Medical School, Boston, MA USA; 2 Center for Alzheimer Research and Treatment, Brigham and Women’s Hospital, Department of Neurology, Brigham and Women’s Hospital, Harvard Medical School, 60 Fenwood Road, Boston, MA 02115 USA

**Keywords:** Alzheimer’s disease, Amyloid, Function, Instrumental activities of daily living, Tau

## Abstract

**Background:**

Emerging difficulty performing cognitively complex everyday tasks, or ‘instrumental activities of daily living’ (IADL) may be an early clinical sign of Alzheimer’s disease (AD). We aimed to investigate how changes over time in everyday functioning relate to cerebral tau burden across the AD clinical spectrum.

**Methods:**

We included 581 participants (73.9 ± 7.6 years old; 52% female) from the Alzheimer’s Disease Neuroimaging Initiative who underwent tau positron emission tomography (PET) and completed at least two assessments of the Functional Activities Questionnaire (FAQ). Participants were classified as cognitively normal (*n* = 334) or symptomatic (*n* = 247). We analyzed the association between longitudinal FAQ scores and baseline tau in six temporal, parietal, and frontal brain regions in mixed-effects models. Models were run in the entire sample, as well as stratified by diagnostic group (cognitively normal or symptomatic). We additionally investigated tau-PET adjusted for, as well as interacting with, amyloid-β.

**Results:**

Greater tau burden in several frontal, temporal, and parietal regions was associated with steeper decline over time in everyday functioning. These findings remained when adjusting for baseline global cortical amyloid-β; amyloid-β itself was only associated with change over time in FAQ scores when tau was not included in the model. When stratifying by diagnostic group, most associations between tau and everyday functioning, adjusted for amyloid-β, were present only in the symptomatic group.

**Conclusions:**

The rate of change in everyday functioning is related to baseline tau burden in various brain regions, more strongly so than global cortical amyloid-β, specifically in cognitively symptomatic individuals. Longitudinal studies in incident dementia populations are needed to better understand functional changes in response to AD pathology across the disease.

**Supplementary Information:**

The online version contains supplementary material available at 10.1186/s13195-023-01267-w.

## Background

Everyday functioning can be represented in “instrumental activities of daily living” (IADL), such as managing finances or preparing meals. Difficulties with performing these cognitively complex activities emerge due to increasing cognitive impairment in Alzheimer’s disease (AD) and related disorders. AD is biologically defined by abnormal levels of amyloid-β and tau [1], which accumulate over decades preceding a dementia diagnosis. Everyday functioning constitutes a clinically relevant outcome, as it reflects an individual’s capacity to live independently. While impairment in everyday functioning traditionally hallmarks the start of the dementia stage, it is known that decline in everyday functioning occurs in prodromal and even preclinical stages of AD [2–4].

Everyday functioning is usually assessed using observer-reported outcome measures that rely on information from someone close to the patient (e.g., a spouse or close friend). An example of such measure is the Functional Activities Questionnaire (FAQ), a brief, ten-item questionnaire that assesses various cognitively complex activities [5], including the ability to assemble tax records or make a cup of coffee. A recent study showed that the FAQ is reliable and strongly correlated with cognitive measures among symptomatic individuals [6], while reliability was somewhat restricted among individuals who are cognitively normal. This was likely due to a limited range in scores in the cognitively normal group. Marshall and colleagues [7] previously identified specific FAQ questions that predicted progression from normal cognition to mild cognitive impairment (MCI).

Comparable to cognitive changes, it is hypothesized that the accumulation of cortical amyloid and cerebral tau underlie deterioration of everyday functioning. Indeed, studies have shown that difficulties performing basic and more cognitively complex activities of daily living have been associated with elevated levels of cortical amyloid-β [8, 9]. Increased cerebral tau has also been associated with early functional changes [10, 11], as have loss of cortical thickness [12] and glucose hypometabolism [13] in various temporal and parietal regions. Finally, tau deposition in frontal regions has also been associated with impairment of everyday functioning in later disease stages [11, 14]. Most of these studies have been cross-sectional and in symptomatic individuals, and less is currently known about the relationship between tau and longitudinal changes in everyday functioning.

In this study, we aimed to investigate how cerebral tau in various regions of interest relates to changes over time in everyday functioning in individuals who are either cognitively normal (CN) or who have been diagnosed with MCI or dementia. We hypothesized that a greater tau burden at baseline would associate with a more rapid decline of everyday functioning over time across diagnostic groups, but more so in symptomatic individuals.

## Methods

### Participants

We selected 633 participants from the Alzheimer’s Disease Neuroimaging Initiative (ADNI; adni.loni.usc.edu) who underwent tau positron emission tomography (PET) scans and who completed at least two functional assessments. Participants were included in ADNI-2 (2011–2016) and ADNI-3 (2016–2022). Participants were categorized as CN, MCI or dementia, based on the ADNI criteria.

We excluded participants with a 15-item Geriatric Depression Scale (GDS; [15]) of ≥ 6 at baseline (*n* = 18) or with missing covariates at baseline (*n* = 34), resulting in a total sample size of 581.

### Tau and amyloid-β PET imaging

Cerebral tau and amyloid-β deposition were assessed using PET scans, with the [^18^F]-flortaucipir (for tau) and [^18^F]-florbetapir or [^18^F]-florbetaben (for amyloid-β) tracers. More details on acquisition and processing methods for these PET scans are described on the ADNI website at https://adni.loni.usc.edu/methods/pet-analysis-method/pet-analysis/.

In brief, for tau images, the mean flortaucipir uptake within each FreeSurfer-defined region of interest (ROI) was calculated and then intensity normalized by the inferior cerebellum reference region. Based on the results of previous studies [10, 11, 14], the ROIs chosen for this investigation were the bilateral entorhinal cortex, inferior temporal, precuneus, posterior cingulate, supramarginal, and the dorsolateral prefrontal (DLPF) composite consisting of the average of the caudal middle frontal, rostral middle frontal, and superior frontal regions.

Amyloid-β PET scans within one year of the baseline tau scan were available for nearly all participants (*n* = 570, 98%). For each tracer separately, an amyloid-β summary standard uptake volume ratio (SUVr) was calculated using a cortical composite and intensity normalized by the whole cerebellum reference region. Continuous values from both tracers were aligned with each other using centiloids, according to the amyloid-β PET processing methods as described in the ADNI study material.

### Measures

We used the 10-item Functional Activities Questionnaire (FAQ) to assess everyday functioning [5]. Each item is rated on a four-point scale, ranging from “normal” to “dependent”. A total score is obtained by summing the items, resulting in a score ranging from 0 to 30 where higher scores represent more functional dependence.

We used overall performance on the five immediate recall trials of the Rey Auditory Verbal Learning Test (RAVLT; [16]) as an assessment of episodic memory functioning. The American National Adult Reading Test (AmNART) was used as a test of premorbid intelligence [17]. Higher scores on the RAVLT and AmNART indicate better performance. Seconds to complete part B of the Trail Making Test (TMT) [18] was used as a measure of mental flexibility, an aspect of executive functioning. Higher scores on the TMT-B represent worse performance.

### Statistical analyses

All analyses were run in R version 4.2.0 [19]. Baseline differences between diagnostic groups were tested using Wilcoxon rank sum test, Pearson’s Chi-squared test, or Fisher’s exact test as appropriate. Correlations between the cognitive tests and FAQ at baseline were computed using Spearman’s *ρ* correlation coefficient.

The associations between baseline tau-PET and amyloid-PET and changes over time in FAQ were investigated using a binomial mixed-effects model. This model was selected to account for dual floor and ceiling effects in the FAQ total score trajectories, as individual trajectories tended to follow a sigmoid curve, asymptoting at the measure’s floor and ceiling with an intermediate period of steep increase. For the dependent variable in the binomial mixed-effect models, we scaled the total FAQ scores by their maximum of 30 so that scores fell between 0 and 1, representing each participant’s score as a proportion of the maximum FAQ score. A logistic sigmoid curve was then to the result. These models were run using the “glmmTMB” package and showed excellent fit. Regular linear mixed models were additionally run for ease of interpretability using the “lmerTest” package and are displayed in the Supplementary Material, though they showed poor fit. First, we analyzed unadjusted change in FAQ over time in the whole sample, as well as stratified by diagnostic group, using random slopes and intercepts by subject. Then, separate models were run analyzing the association between longitudinal FAQ scores and the interaction of time with (1) baseline tau-PET, (2) baseline amyloid-PET, and (3) tau-PET adjusted for amyloid-PET. These models included tau-PET and/or amyloid-PET by time interactions. For models involving tau-PET, a separate model was run for each ROI. For models including centiloids, one individual with an outlying centiloid value was excluded. Reported model *p*-values are adjusted for multiple comparisons using the False Discovery Rate (FDR) method. All models were adjusted for baseline age, gender, and the interaction of baseline age with time. We additionally adjusted models for baseline RAVLT, AmNART, TMT-B, and determined that adding cognitive functioning improved model fits as based on the Akaike Information Criterion.

For secondary analyses, we predicted longitudinal FAQ from tau-PET with an amyloid-PET adjustment in the CN and symptomatic groups separately. In CN only models, one outlying individual was excluded from analyses, because their FAQ score increased rapidly from no impairment to severe impairment at their second follow-up visit. Additionally, we examined the three-way interaction between tau-PET, amyloid-PET, and time in predicting the FAQ total score within the full sample. All models used the same random-effects structure and covariates as above.

Sensitivity analyses were run with a grouped version of the amyloid-PET measure, with Aβ+ indicating amyloid-β levels elevated above a threshold (1.11 SUVr for [^18^F]-florbetapir or 1.08 SUVr for [^18^F]-florbetaben) and Aβ- indicating not elevated.

## Results

We included 581 participants (73.9 ± 7.6 years old, 52% female) who were either CN (*n* = 334, 57%) or had a diagnosis of MCI (*n* = 191, 33%) or Alzheimer’s disease dementia (AD, *n* = 56, 10%). These last two groups were combined into a ‘symptomatic’ group. Symptomatic participants were older, more often male and had fewer years of education, compared to the CN group. Demographic characteristics are displayed in Table 1.

**Table 1:** Baseline demographics and characteristics

	**All**	**Cognitively normal**	**Symptomatic**	**P-value**
**N (%)**	581 (100.0)	334 (57.5)	247 (42.5)	
**Age in years**	73.9 ± 7.6	72.9 ± 7.2	75.1 ± 8.0	< 0.001
**Female, n (%)**	304 (52.3)	201 (60.2)	103 (41.7)	< 0.001
**Years of education**	16.5 ± 2.5	16.7 ± 2.3	16.1 ± 2.7	0.024
**Race, n (%)**				0.220
White	534 (91.9)	300 (89.9)	234 (94.7)	
Black, African American	25 (4.3)	18 (5.4)	7 (2.8)	
Asian	8 (1.4)	6 (1.8)	2 (0.8)	
American Indian, Alaskan Native	2 (0.3)	2 (0.6)	0 (0.0)	
Multiracial	11 (1.9)	8 (2.4)	3 (1.2)	
**Hispanic/Latino Ethnicity, n (%)**	27 (4.6)	19 (5.7)	8 (3.2)	0.164
**Baseline FAQ**	2.4 ± 5.0	0.2 ± 0.9	5.3 ± 6.6	< 0.001
**MMSE**	28.2 ± 2.3	29.1 ± 1.2	27.0 ± 2.8	< 0.001
**AmNART VIQ**	119.9 ± 10.9	121.7 ± 10.3	117.4 ± 11.3	< 0.001
**Amyloid-PET**				
Centiloids	32.1 ± 40.8	23.3 ± 31.7	44.3 ± 48.1	< 0.001
Positive, n (%)	259 (44.6)	125 (37.4)	134 (54.3)	< 0.001
**Tau-PET**				
Entorhinal	1.22 ± 0.22	1.15 ± 0.13	1.32 ± 0.28	< 0.001
Inferior temporal	1.29 ± 0.27	1.22 ± 0.14	1.38 ± 0.36	< 0.001
Precuneus	1.17 ± 0.21	1.12 ± 0.10	1.23 ± 0.29	< 0.001
Posterior cingulate	1.14 ± 0.17	1.11 ± 0.10	1.18 ± 0.23	< 0.001
Supramarginal	1.12 ± 0.17	1.09 ± 0.10	1.17 ± 0.23	< 0.001
DLPF	1.07 ± 0.17	1.04 ± 0.09	1.11 ± 0.24	0.002
**Follow-up duration in years**	2.5 ± 1.2	2.6 ± 1.0	2.5 ± 1.3	0.067

Data are displayed as mean ± standard deviation, except as stated otherwise

*Abbreviations: AmNART* American National Adult Reading Test, *DLPF* Dorsolateral prefrontal cortex, *FAQ* Functional Activities Questionnaire, *MMSE* Mini-Mental State Examination, *PET* Positron emission tomography, *VIQ* Verbal intelligence quotient

At baseline, the overall average FAQ score was 2.4 ± 5.0. CN participants had a significantly lower FAQ at baseline of 0.2 ± 0.9, compared to 5.3 ± 6.6 among symptomatic participants (*p* < 0.001). Baseline FAQ total scores were correlated with measures of executive functioning (Spearman’s *ρ* = 0.46, *p* < 0.001) and episodic memory (*ρ* = -0.50, *p* < 0.001), as well as premorbid intelligence (*ρ* = -0.25, *p* < 0.001). These correlations were less evident in the CN participants, with only the executive functioning task showing a significant correlation (*ρ* = 0.16, *p* = 0.003; both others *p* > 0.05).

Overall, unadjusted FAQ scores showed no significant change over time (odds ratio (OR) = 0.98, 95% confidence interval (95%CI) = [0.83, 1.16], *p* = 0.843). When stratifying by diagnostic group, it appeared CN participants’ odds for increasing FAQ were not elevated (OR = 1.01, 95%CI = [0.60, 1.69], *p* = 0.976), while participants who were symptomatic had higher odds for increasing FAQ scores over time (OR = 1.24, 95%CI = [1.12, 1.37], *p* < 0.001), representing a 24% increase in their degree of impairment as measured across FAQ items.

**Fig. 1:**
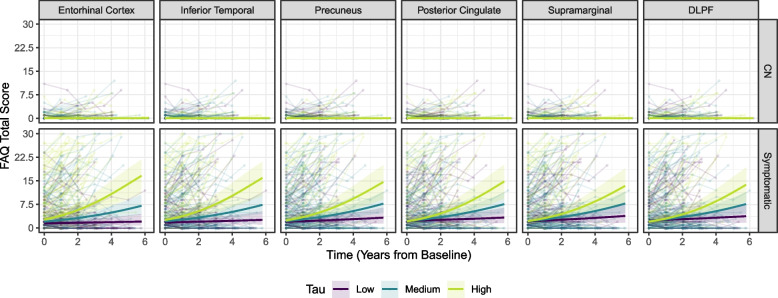
Association between tau and change in FAQ scores over time, stratified by diagnostic group

Including adjustments for baseline cognitive performance altered the estimates for change in everyday functioning over time only marginally; therefore, only the adjusted models are reported below. Models not adjusted for baseline cognitive performance are displayed in the Supplementary Material. Tau accumulation in all investigated ROIs was associated with an increase in FAQ scores over time in both cognitively normal and symptomatic individuals combined (all *p* < 0.001). This association remained significant in all brain regions (all *p* < 0.001) after adjusting for amyloid-PET. Among CN participants, tau in the entorhinal and inferior temporal cortex was significantly associated with changes on the FAQ, but only tau in the inferior temporal cortex remained significantly associated with FAQ changes when adding amyloid to the model (*p* = 0.035; all other *p* > 0.05). In symptomatic individuals, we observed a significant association between baseline tau-PET in all ROIs and change on the FAQ, both without and with adjustment for amyloid. All estimates are displayed in Table 2 and associations for the amyloid-adjusted models are visualized in Fig. 1.

**Table 2:** Association between baseline amyloid-PET and tau-PET and change in FAQ scores over time, in binomial models

	**All**		**Cognitively normal**		**Symptomatic**	
	OR [95%CI]	*p*	OR [95%CI]	*p*	OR [95%CI]	*p*
**Model 1: only amyloid-PET**						
Centiloids	1.00 [1.00, 1.01]	< 0.001	1.00 [1.00, 1.01]	0.474	1.00 [1.00, 1.01]	< 0.001
**Model 2: only tau-PET**						
Entorhinal	2.65 [1.92, 3.66]	< 0.001	5.60 [1.35, 23.23]	0.035	2.35 [1.72, 3.21]	< 0.001
Inferior temporal	2.21 [1.72, 2.84]	< 0.001	5.02 [1.46, 17.25]	0.022	2.00 [1.57, 2.55]	< 0.001
Precuneus	2.36 [1.73, 3.22]	< 0.001	4.86 [0.77, 30.59]	0.162	2.12 [1.58, 2.85]	< 0.001
Posterior cingulate	3.07 [2.09, 4.51]	< 0.001	2.29 [0.03, 13.83]	0.474	2.82 [1.95, 4.09]	< 0.001
Supramarginal	2.71 [1.86, 3.95]	< 0.001	6.27 [1.03, 38.30]	0.085	2.39 [1.66, 3.43]	< 0.001
DLPF	2.85 [1.95, 4.14]	< 0.001	3.17 [0.31, 32.13]	0.448	2.62 [1.83, 3.74]	< 0.001
**Model 3: tau-PET, adjusted for amyloid-PET**						
Entorhinal	2.38 [1.62, 3.49]	< 0.001	4.93 [1.06, 22.99]	0.081	2.04 [1.40, 2.99]	< 0.001
Inferior temporal	1.93 [1.47, 2.55]	< 0.001	4.53 [1.31, 15.66]	0.035	1.73 [1.32, 2.28]	< 0.001
Precuneus	1.94 [1.39, 2.71]	< 0.001	2.89 [0.39, 21.20]	0.423	1.79 [1.30, 2.47]	< 0.001
Posterior cingulate	2.52 [1.68, 3.77]	< 0.001	1.58 [0.24, 10.35]	0.701	2.37 [1.60, 3.51]	< 0.001
Supramarginal	2.09 [1.40, 3.13]	< 0.001	3.37 [0.49, 23.31]	0.336	1.91 [1.29, 2.83]	0.003
DLPF	2.22 [1.50, 3.30]	< 0.001	1.34 [0.12, 15.43]	0.843	2.14 [1.46, 3.12]	< 0.001
**Model 4: tau-PET by amyloid-PET by time three-way interaction**						
Entorhinal	1.00 [0.99, 1.00]	0.444	0.99 [0.95, 1.03]	0.591	1.00 [0.99, 1.00]	0.423
Inferior temporal	1.00 [0.99, 1.00]	0.423	0.98 [0.94, 1.01]	0.336	1.00 [0.99, 1.00]	0.618
Precuneus	0.99 [0.99, 1.00]	0.083	0.98 [0.94, 1.03]	0.626	0.99 [0.99, 1.00]	0.192
Posterior cingulate	1.00 [0.99, 1.01]	0.618	0.98 [0.94, 1.03]	0.618	1.00 [0.99, 1.01]	0.713
Supramarginal	1.00 [0.99, 1.01]	0.773	0.98 [0.93, 1.03]	0.474	1.00 [0.99, 1.01]	0.880
DLPF	0.99 [0.98, 1.00]	0.300	0.98 [0.92, 1.05]	0.652	0.99 [0.99, 1.00]	0.336

The outcome variable for all models is the degree of impairment measured as the proportion of the maximum attainable score on the FAQ (total score / 30). Displaying amyloid-PET or tau-PET by time interactions. P-values are corrected for multiple testing. All models are adjusted for age, gender, the interaction of age with time, RAVLT, AmNART and TMT-B

*Abbreviations: DLPF* Dorsolateral prefrontal cortex, *FAQ* Functional Activities Questionnaire, *OR* Odds ratio, *95%CI* 95% confidence interval

We additionally ran models with an interaction between tau, amyloid, and time. These three-way interactions were not significant, neither in the whole group nor in the diagnostic groups (see Table 2).

In sensitivity analyses with dichotomized amyloid, results remained the same. For ease of interpretability, we also ran all above binomial models as linear models. Linear models had suboptimal fit, but results were comparable to the binomial models, except that tau in the precuneus and supramarginal cortex was also associated with change in FAQ over time. These associations disappeared when including amyloid-PET in the model. All sensitivity analyses are shown in the Supplementary Material.

Figure 1 displays the association between change in FAQ scores over time and baseline tau accumulation in the ROIs, stratified for diagnostic group. For visualization purposes, we divided tau-PET into three groups, representing the mean tau-PET level (medium) and one standard deviation below (low) and above (high) the mean. The curves are adjusted for amyloid, with age, gender, RAVLT, TMT-B, AmNART, and the interaction of age with time as covariates.

## Discussion

In this study, we demonstrated that baseline levels of tau in various cerebral regions were associated with decline over time in cognitively complex activities of daily living, as measured by the FAQ. In individuals who were initially cognitively normal, baseline tau in the entorhinal and inferior temporal regions was associated with decline in everyday functioning over time, even when adjusting for global amyloid-β burden. In symptomatic individuals, tau in all investigated areas was related to future decline in everyday functioning, even when accounting for amyloid-β.

On average, everyday functioning declined over time in the entire sample. As expected, the change in everyday functioning was larger in symptomatic than in CN individuals. In fact, everyday functioning only marginally changed among individuals who were CN at inclusion. It has been described previously that everyday functioning as measured using observer-reported outcome measures may decline in preclinical and prodromal disease stages [3, 20–23]. It is possible that, with a more extensive and fine-grained instrument and/or longer follow-up times, we would have been able to capture even more changes in everyday functioning.

Nevertheless, our findings show a relationship between everyday functioning and cerebral tau in temporal, parietal, and frontal regions both in symptomatic and CN individuals. Various previous studies have shown a cross-sectional relationship between tau and everyday functioning, most evidently in temporal, frontal and parietal brain regions [10, 11, 14, 24], which informed our selection of regions to investigate in the present study. However, the association between longitudinal changes in everyday functioning and tau is less well-studied. Here, we demonstrate that those who are cognitively symptomatic and who have higher (i.e., more abnormal) levels of tau in several (medial) temporal, frontal, and parietal brain regions showed more progression of difficulties with everyday functioning over an average time of two-and-a-half years. Elevated levels of tau in the entorhinal and inferior temporal cortices were even related to a decline in everyday functioning in cognitively normal individuals, when not considering global amyloid-β burden. Even when accounting for global amyloid-β, tau in the inferior temporal cortex was related to a greater change in everyday functioning. The regional differences between cognitively normal and symptomatic individuals can be explained by the fact that the latter group is in a later disease stage. As AD progresses, brain pathology spreads through the cortex. Symptomatic individuals have more cognitive—and functional—impairments. As such, we were more likely to find associations in more brain regions in symptomatic individuals. Temporal regions are known to accumulate tau, but not necessarily amyloid-β, in early disease stages [25]. This might explain why we found a relationship between inferior temporal tau and functional change even in individuals in an early disease stage.

Regarding global cortical amyloid, only cognitively symptomatic participants with more abnormal levels of amyloid showed more decline of everyday functioning. We did not observe such a relationship in CN individuals, which might be explained by either the lower levels of amyloid, or the limited changes in everyday functioning in this group. We further attempted to model the interaction between tau and amyloid in the change in everyday functioning and found that elevated global cortical amyloid and elevated tau in the entorhinal, inferior temporal, posterior cingulate and supramarginal cortices predicted more decline in everyday functioning in the entire sample. Hanseeuw and colleagues [26] previously demonstrated a sequential relationship between amyloid, tau, and cognition, where those with higher levels of amyloid and more increase in tau became cognitively impaired. It is conceivable that a similar process underlies impairment in everyday functioning. Our results suggest an interplay between the two proteins in subsequent functional decline, although our study did not include repeated measures of amyloid or tau. It should be noted that the combined effect of elevated amyloid and tau was visible only the total group, and not in either the CN or symptomatic subgroups separately. A potential explanation for this is that the two subgroups may have been too small to support the modeling of complex interactions of variables. The lack of sufficient variability in levels of amyloid, tau, or everyday functioning in either group alone may be another possible reason for the instability of this finding.

Everyday functioning is conceptually close to cognitive functioning, as the performance of complex activities relies on various cognitive functions [27]. Therefore, our findings track with previous studies showing that tau abnormalities are related to cognitive impairment [28], and more strongly so than amyloid [29]. That said, our findings that the estimates of change in everyday functioning were not altered substantially when adjusting for baseline cognitive performance led us to believe that there is unique functional change in relation to tau and amyloid that is not explained by performance on cognitive tests. This may imply that function is not simply a proxy of cognition in everyday life. More studies are needed where both everyday functioning and cognitive test performance are measured repeatedly to better understand how cognitive and functional changes relate to one another in the context of AD.

We made use of the FAQ in this study, which is a brief questionnaire that has recently been shown to have good reliability and validity [6, 30]. Further, it seems to have limited bias for education, ethnicity, race, language, and sex [30]. An advantage of the FAQ is that it comprises only ten questions and therefore poses very little burden on the person completing it. However, we observed a substantial floor effect in CN individuals, with relatively little change over two-and-a-half years. More sensitive measures of IADL performance may be capable of capturing more subtle changes, which seems particularly relevant in early disease stages. Performance-based and digital tools in particular have great potential to provide fine-grained measurements of objective IADL performance at early stages of AD.

### Limitations

This study had a few limitations. First, the follow-up duration was relatively short, with an average of two-and-a-half years. Especially in CN individuals, notable changes in IADL may take longer to evolve. Second, our sample was mostly comprised of relatively highly educated White individuals, limiting the generalizability of our findings to underrepresented groups. Third, the IADL measure we used, the FAQ, had both floor and ceiling effects in the current sample, though the binomial model accounted for this effect. Strengths of this study included the availability of regional tau measures in several relevant brain areas, as well as repeated assessments of IADL performance for a relatively large longitudinal imaging cohort.

## Conclusion

In conclusion, decline in everyday functioning in AD seems to be driven by tau in various areas of the temporal, parietal, and frontal lobes, especially in mildly cognitively symptomatic individuals. Future studies should further investigate the causal role of tau in the performance of complex everyday activities, including repeated measures of tau and more sensitive measures of IADL in more diverse samples.

## Supplementary Information

**Additional file 1.**

## Data Availability

The datasets used and/or analyzed during the current study are available through the LONI Image and Data Archive repository, upon registration via https://adni.loni.usc.edu/.

## Abbreviations

AD: Alzheimer’s disease
ADNI: Alzheimer’s Disease Neuroimaging Initiative
AmNART: American National Adult Reading Test
CN: Cognitively normal
DLPF: Dorsolateral prefrontal cortex
FAQ: Functional Activities Questionnaire
FDR: False Discovery Rate
GDS: Geriatric Depression Scale
IADL: Instrumental activities of daily living
LMM: Linear mixed model
MCI: Mild cognitive impairment
MMSE: Mini-Mental State Examination
OR: Odds ratio
RAVLT: Rey Auditory Verbal Learning Test
ROI: Region of interest
SUVr: Standard uptake volume ratio
TMT: Trail Making Test
VIQ: Verbal intelligence quotient
95%CI: 95% Confidence interval
