# Supplementary material for: Regional cerebral tau predicts decline in everyday functioning across the Alzheimer’s disease spectrum
Source: Alzheimers Res Ther. 2023 Jul 5;15:120. doi: 10.1186/s13195-023-01267-w (PMC10320884; doi:10.1186/s13195-023-01267-w)
Supplement: Supplementary file 1 — Additional file 1. [file 13195_2023_1267_MOESM1_ESM.docx]

Supplementary Table 1. Association between baseline amyloid and tau and change in FAQ scores over time, in binomial models, not adjusted for baseline cognitive performance.

|  | All | | Cognitively normal | | Symptomatic | |
| --- | --- | --- | --- | --- | --- | --- |
|  | OR [95%CI] | p | OR [95%CI] | p | OR [95%CI] | p |
| Model 1: only amyloid-PET  Global | 1.00 [1.00, 1.01] | <0.001 | 1.00 [1.00, 1.01] | 0.494 | 1.00 [1.00, 1.01] | <0.001 |
| Model 2: only tau-PET  Entorhinal  Inferior temporal  Precuneus  Posterior cingulate  Supramarginal  dlpf | 2.77 [1.98, 3.87]  2.32 [1.78, 3.01]  2.63 [1.89, 3.66]  3.40 [2.27, 5.09]  3.04 [2.04, 4.52]  3.11 [2.10, 4.62] | <0.001  <0.001  <0.001  <0.001  <0.001  <0.001 | 5.53 [1.33, 22.91]  4.89 [1.42, 16.83]  4.75 [0.75, 29.90]  2.23 [0.37, 13.59]  6.13 [1.00, 37.63]  3.06 [0.30, 31.44] | 0.037  0.025  0.167  0.496  0.092  0.483 | 2.42 [1.76, 3.33]  2.05 [1.61, 2.62]  2.21 [1.63, 3.00]  2.94 [2.01, 4.30]  2.51 [1.73, 3.64]  2.71 [1.88, 3.92] | <0.001  <0.001  <0.001  <0.001  <0.001  <0.001 |
| Model 3: tau-PET, adjusted for amyloid-PET  Entorhinal  Inferior temporal  Precuneus  Posterior cingulate  Supramarginal  dlpf | 2.52 [1.70, 3.72]  2.02 [1.52, 2.69]  2.10 [1.48, 2.97]  2.67 [1.76, 4.06]  2.25 [1.49, 3.41]  2.34 [1.56, 3.51] | <0.001  <0.001  <0.001  <0.001  <0.001  <0.001 | 4.94 [1.06, 22.99]  4.45 [1.28, 15.44]  2.85 [0.39, 21.00]  1.55 [0.24, 10.24]  3.33 [0.48, 23.21]  1.28 [0.11, 15.03] | 0.080  0.037  0.456  0.709  0.357  0.860 | 2.11 [1.44, 3.10]  1.78 [1.35, 2.35]  1.85 [1.33, 2.56]  2.42 [1.62, 3.62]  1.97 [1.32, 2.94]  2.19 [1.49, 3.22] | <0.001  <0.001  <0.001  <0.001  0.002  <0.001 |
| Model 4: tau-PET by amyloid-PET by time three-way interaction  Entorhinal  Inferior temporal  Precuneus  Posterior cingulate  Supramarginal  dlpf | 1.00 [0.99, 1.00]  1.00 [0.99, 1.00]  0.99 [0.99, 1.00]  1.00 [0.99, 1.01]  1.00 [0.99, 1.01]  1.00 [0.99, 1.00] | 0.567  0.527  0.137  0.785  0.981  0.456 | 0.99 [0.95, 1.03]  0.98 [0.94, 1.01]  0.99 [0.94, 1.03]  0.99 [0.94, 1.03]  0.98 [0.93, 1.03]  0.98 [0.92, 1.05] | 0.587  0.337  0.648  0.640  0.494  0.668 | 1.00 [0.99, 1.00]  1.00 [0.99, 1.00]  0.99 [0.99, 1.00]  1.00 [0.99, 1.01]  1.00 [0.99, 1.01]  0.99 [0.99, 1.00] | 0.483  0.652  0.245  0.785  0.785  0.394 |

*Note:* Displaying amyloid or tau by time interactions. P-values are corrected for multiple testing. All models are adjusted for age, gender, and the interaction of age with time.

*Abbreviations:* DLPF, dorsolateral prefrontal cortex; FAQ, Functional Activities Questionnaire; OR, odds ratio; 95%CI, 95% confidence interval.

Supplementary Table 2. Association between dichotomized baseline amyloid and tau and change in FAQ scores over time, in binomial models.

|  | All | | Cognitively normal | | Symptomatic | |
| --- | --- | --- | --- | --- | --- | --- |
|  | OR [95%CI] | p | OR [95%CI] | p | OR [95%CI] | p |
| Model 1: only amyloid-PET group  Global | 1.40 [1.18, 1.65] | <0.001 | 1.17 [0.76, 1.79] | 0.699 | 1.44 [1.20, 1.73] | <0.001 |
| Model 2: tau, adjusted for amyloid-PET group  Entorhinal  Inferior temporal  Precuneus  Posterior cingulate  Supramarginal  dlpf | 2.41 [1.66, 3.49]  1.97 [1.50, 2.58]  2.02 [1.46, 2.79]  2.61 [1.75, 3.91]  2.20 [1.48, 3.26]  2.32 [1.58, 3.43] | <0.001  <0.001  <0.001  <0.001  <0.001  <0.001 | 4.65 [1.10, 19.69]  4.46 [1.33, 15.00]  3.09 [0.44, 21.63]  1.73 [0.28, 10.79]  3.58 [0.53, 24.45]  1.42 [0.12, 16.93] | 0.090  0.041  0.433  0.725  0.357  0.873 | 2.08 [1.44, 3.01]  1.77 [1.36, 2.30]  1.85 [1.36, 2.51]  2.44 [1.65, 3.59]  1.99 [1.36, 2.91]  2.21 [1.53, 3.20] | <0.001  <0.001  <0.001  <0.001  0.001  <0.001 |
| Model 3: tau-PET by amyloid-PET group by time three-way interaction  Entorhinal  Inferior temporal  Precuneus  Posterior cingulate  Supramarginal  dlpf | 0.77 [0.29, 2.01]  0.36 [0.13, 1.05]  0.39 [0.10, 1.50]  0.97 [0.27, 3.46]  1.01 [0.25, 4.08]  0.44 [0.10, 1.92] | 0.740  0.143  0.349  0.983  0.985  0.450 | 1.97 [0.10, 37.36]  0.47 [0.04, 5.40]  1.55 [0.02, 99.81]  2.48 [0.05, 116.34]  0.29 [0.01, 13.51]  0.62 [0.00, 93.06] | 0.771  0.725  0.897  0.771  0.725  0.897 | 0.57 [0.20, 1.64]  0.32 [0.08, 1.35]  0.38 [0.09, 1.59]  0.83 [0.21, 3.20]  2.16 [0.42, 11.02]  0.40 [0.09, 1.83] | 0.465  0.262  0.357  0.873  0.530  0.418 |

*Note:* Displaying amyloid or tau by time interactions. P-values are corrected for multiple testing.

*Abbreviations:* DLPF, dorsolateral prefrontal cortex; FAQ, Functional Activities Questionnaire; OR, odds ratio; 95%CI, 95% confidence interval.

Supplementary Table 3. Association between continuous baseline amyloid and tau and change in FAQ scores over time, in linear models.

|  | All | | Cognitively normal | | Symptomatic | |
| --- | --- | --- | --- | --- | --- | --- |
|  | B [95%CI] | p | B [95%CI] | p | B [95%CI] | p |
| Model 1: FAQ, unadjusted  Time | 0.59 [0.45, 0.73] | <0.001 | 0.08 [0.02, 0.14] | 0.014 | 1.26 [0.96, 1.56] | <0.001 |
| Model 2: only amyloid-PET  Global | 0.01 [0.01, 0.02] | <0.001 | 0.00 [0.00, 0.00] | 0.604 | 0.02 [0.01, 0.02] | <0.001 |
| Model 3: only tau-PET  Entorhinal  Inferior temporal  Precuneus  Posterior cingulate  Supramarginal  dlpf | 3.69 [3.07, 4.32]  3.31 [2.76, 3.86]  3.72 [3.03, 4.42]  4.30 [3.46, 5.15]  3.94 [3.11, 4.78]  4.24 [3.38, 5.10] | <0.001  <0.001  <0.001  <0.001  <0.001  <0.001 | 0.77 [0.31, 1.24]  0.73 [0.32, 1.14]  0.83 [0.21, 1.46]  0.47 [-0.14, 1.08]  0.99 [0.38, 1.61]  0.64 [-0.08, 1.36] | 0.002  0.001  0.015  0.174  0.003  0.117 | 3.84 [2.77, 4.91]  3.44 [2.56, 4.32]  3.71 [2.63, 4.78]  4.84 [3.48, 6.20]  4.26 [2.92, 5.59]  4.53 [3.21, 5.85] | <0.001  <0.001  <0.001  <0.001  <0.001  <0.001 |
| Model 4: tau-PET, adjusted for amyloid-PET  Entorhinal  Inferior temporal  Precuneus  Posterior cingulate  Supramarginal  dlpf | 3.43 [2.68, 4.17]  2.96 [2.34, 3.58]  3.21 [2.45, 3.97]  3.70 [2.79, 4.60]  3.21 [2.30, 4.12]  3.53 [2.60, 4.46] | <0.001  <0.001  <0.001  <0.001  <0.001  <0.001 | 0.71 [0.23, 1.19]  0.70 [0.29, 1.12]  0.57 [-0.08, 1.22]  0.33 [-0.29, 0.95]  0.70 [0.06, 1.34]  0.34 [-0.41, 1.09] | 0.007  0.002  0.118  0.374  0.050  0.448 | 3.35 [2.03, 4.68]  2.95 [1.93, 3.98]  3.08 [1.90, 4.26]  4.13 [2.68, 5.59]  3.39 [1.93, 4.85]  3.74 [2.31, 5.16] | <0.001  <0.001  <0.001  <0.001  <0.001  <0.001 |
| Model 5: tau-PET by amyloid-PET by time three-way interaction  Entorhinal  Inferior temporal  Precuneus  Posterior cingulate  Supramarginal  dlpf | 0.02 [0.00, 0.04]  0.01 [0.00, 0.03]  0.00 [-0.02, 0.01]  0.02 [0.00, 0.04]  0.02 [0.00, 0.04]  0.01 [-0.01, 0.03] | 0.019  0.059  0.860  0.056  0.024  0.374 | 0.00 [-0.02, 0.01]  -0.01 [-0.02, 0.00]  0.00 [-0.02, 0.01]  0.00 [-0.02, 0.01]  -0.01 [-0.03, 0.01]  -0.01 [-0.03, 0.01] | 0.813  0.118  0.622  0.673  0.427  0.364 | 0.00 [-0.03, 0.03]  0.00 [-0.02, 0.02]  -0.01 [-0.04, 0.01]  0.00 [-0.03, 0.03]  0.01 [-0.02, 0.04]  -0.01 [-0.04, 0.02] | 0.944  0.944  0.371  0.845  0.523  0.584 |

*Note:* Displaying amyloid or tau by time interactions. P-values are corrected for multiple testing.

*Abbreviations:* DLPF, dorsolateral prefrontal cortex; FAQ, Functional Activities Questionnaire; 95%CI, 95% confidence interval.
